# Association between sleep quality, depression, and quality of Life in older adults

**DOI:** 10.1192/j.eurpsy.2026.11587

**Published:** 2026-07-31

**Authors:** N. Bouattour, S. Omri, N. Smaoui, I. Gassara, M. Mâalej, M. Mâalej, R. Feki, N. charfi, L. Zouari

**Affiliations:** Psychiatry C, Hedi Chaker University Hospital, Sfax, Tunisia

## Abstract

**Introduction:**

Sleep quality, depression, and quality of life (QoL) are closely interconnected, especially in the elderly population, where these factors significantly impact health and well-being.

**Objectives:**

Our study aimed to investigate the potential relationships between sleep quality, depression, and quality of life (QoL) in an elderly population.

**Methods:**

Our study was a cross-sectional, descriptive, and analytical design conducted over a period of five months from July 2024 to November 2024. It included patients aged 65 and older who consulted general practitioners at two local clinics in the Sfax region. Data collection was carried out using a pre-established form. Sleep disorders were assessed using the validated Arabic version of the Pittsburgh Sleep Quality Index (PSQI) and the Arabic version of the Epworth Sleepiness Scale (ESS). Depression was assessed using the Geriatric Depression Scale (GDS-15). QoL was assessed using the SF-12 scale in its validated Arabic version.

**Results:**

Sixty patients participated in our study. The median age was 70 years [IQR (67-76.5)]. The sex ratio (M/F) was 0.7. The median overall PSQI score was 13 [11-15]. Sleep quality was poor in 65% of participants. An ESS score >10 was found in 90% of patients. The median ESS score was 13 [IQR (11-14)]. The prevalence of depression was 35%. The median GDS-15 score was 9 [IQR (6-13)]. The median physical health dimension score was 24.01 [IQR (21.3-28.46)] and the mental health dimension score was 44.54 [IQR (29.34-51.77)]. The overall PSQI score was correlated with the Epworth sleep score (p=0.040), the depression score (p=0.02, r=0.389), and the physical health dimension of the QoL scale (p=0.0001, r=-0.511). Depression was also associated with impaired mental health (p=0.0001, r=-0.653).

**Conclusions:**

This study highlights significant correlations between poor sleep quality, depression, and impaired QoL in older adults. These findings underscore the importance of comprehensive care that includes screening and treatment for sleep disorders and depression to improve the well-being of seniors.

**Disclosure of Interest:**

None Declared

